# Molecular investigation and viral load analysis of bovine respiratory syncytial virus in cattle with bovine respiratory disease complex in Inner Mongolia, China

**DOI:** 10.3389/fcimb.2026.1720214

**Published:** 2026-02-13

**Authors:** Yaxing Ban, Fan Zhang, Zhidan Zhang, Ligeer Qi, Chunxia Chai, Ninigen Xi, Weiguang Zhou

**Affiliations:** 1 College of Veterinary Medicine, Inner Mongolia Agricultural University, Hohhot, China; 2 Key Laboratory of Clinical Diagnosis and Treatment Technology in Animal Disease, Ministry of Agriculture, Hohhot, China; 3 Veterinary Research Institute, Inner Mongolia Academy of Agricultural & Animal Husbandry Sciences, Hohhot, China; 4 Tongliao Municipal Administration of Government Services and Data Management, Tongliao, China

**Keywords:** bovine respiratory disease complex, bovine respiratory syncytial virus, epidemiological investigation, multiplex real-time PCR, viral load

## Abstract

Bovine respiratory syncytial virus (BRSV) is a major pathogen of the bovine respiratory disease complex (BRDC), causing significant morbidity and economic losses worldwide. However, its molecular epidemiology in Inner Mongolia, one of China’s largest cattle-producing regions, remains poorly characterized, particularly among calves with clinical respiratory disease. In this study, we developed and performed a preliminary evaluation of a multiplex RT-qPCR assay targeting the F and N genes of BRSV, with ABL1 as an endogenous control, for rapid and reliable detection in symptomatic calves. A total of 909 clinical samples collected in 2023 from calves showing respiratory signs were tested, revealing an overall BRSV detection rate of 21.23% among clinically affected calves with respiratory disease. Lung tissues showed higher detection rates (35.43%) than nasal swabs (17.85%). The virus was more frequently detected in central and western regions, and its occurrence exhibited seasonal peaks in summer and winter. Intensive and large-scale farms tended to have higher infection rates and viral loads than pastoral households. Viral load also varied by sample type and season, being highest in lung tissues and during spring–summer. These findings provide baseline molecular epidemiological data on BRSV among clinically affected calves in Inner Mongolia and, although they are not representative of population-level prevalence, they highlight the burden of BRSV detection within this symptomatic diagnostic cohort and suggest that the developed multiplex RT-qPCR assay may serve as a useful tool for diagnostic screening and targeted monitoring in clinically affected herds or outbreak investigations.

## 1 Introduction

Bovine respiratory syncytial virus (BRSV) is a significant viral pathogen associated with the bovine respiratory disease complex (BRDC), playing a pivotal role in the pathogenesis and progression of this disease (Chang et al., 2022; Ferella et al., 2023). BRSV infection not only severely impacts animal welfare and the economic viability of livestock farming but also substantially increases the risk of secondary bacterial infections (Johnson et al., 2022; Kamel et al., 2024). Since its identification in Europe in the 1970s, BRSV has become widespread globally (Paccaud and Jacquier, 1970). The virus markedly enhances the susceptibility of calves to secondary infections (Johnston et al., 2019; Martínez et al., 2023), promotes bacterial colonization in the lower respiratory tract (Sudaryatma et al., 2018; Sudaryatma et al., 2020), and can persist for extended periods in the natural host, the cow (Paccaud and Jacquier, 1970). Outbreaks occur most frequently in recently weaned calves and young cattle within intensive farming systems, where morbidity rates can reach 60% and mortality rates approximate 20%, resulting in significant economic losses (Hou et al., 2024).

BRSV belongs to the *Orthopneumovirus* genus of the family *Pneumoviridae* and is an enveloped, non-segmented, minus-strand RNA virus (Stott and Taylor, 1985; Chang et al., 2022). The total length of the genome is approximately 15 kb and encodes a variety of structural and non-structural proteins. Among these, the fusion glycoprotein (F), which consists of 574 amino acid residues, is responsible for mediating the fusion of the virus with the host cell membrane. It is highly conserved across different isolates (Makoschey and Berge, 2021). The nucleoprotein (N), comprising 391 amino acid residues, is also highly conserved and is present in large quantities in both viruses and infected cells (Valarcher et al., 2000). These characteristics render the F and N genes common targets for qPCR detection. The inclusion of an internal control gene in qPCR is an essential monitoring measure (Cheng et al., 2022). The proto-oncogene ABL1 is widely present in biological systems and exhibits high sequence conservation. Compared to GAPDH, ABL1 demonstrates greater stability (Li et al., 2024), making it a suitable internal control gene for molecular detection methods.

The epidemic dynamics of BRSV are influenced by host immunity (Da Silva Barcelos et al., 2024), environmental conditions (Stokstad et al., 2020), population density, and management measures (Norström et al., 2000; Gorden and Plummer, 2010), exhibiting seasonal and local epidemic characteristics (Yamaguchi et al., 2021). Epidemiological surveillance utilizing molecular detection not only facilitates the early identification of epidemics but also provides a foundation for analyzing transmission routes and formulating prevention and control strategies. Reverse transcription quantitative polymerase chain reaction (RT-qPCR) has emerged as a preferred method for the detection of BRSV and other common respiratory pathogens due to its high sensitivity and specificity (Loy et al., 2018; Hao et al., 2024).

However, most current RT-qPCR assays for BRSV rely on a single viral target gene, which may, in principle, compromise detection reliability when sequence changes occur within the short amplicon region, or when target-specific assay performance varies across specimens. Inner Mongolia Autonomous Region is a significant livestock breeding base in China, ranking high in cattle population. Although there have been reported outbreaks of BRSV in different provinces within China (Guo et al., 2021; Chang et al., 2022; Zhou et al., 2023), systematic molecular epidemiological data for this vast region with diverse livestock rearing patterns remain limited.

Most current diagnostic methods for BRSV rely on single-gene detection of only one conserved viral component. While this strategy simplifies assay design and interpretation, it also means that minor sequence changes within the selected amplicon could, in principle, reduce analytical sensitivity and, potentially leading to false-negative results (Sudaryatma et al., 2020). In China, the diagnostic standards and published assays also predominantly adopt a single-gene strategy. For example, the Jilin Provincial Standard DB22/T 3473–2023 and the Chinese Veterinary Medical Association (CVMA) Group Standard T/CVMA 145–2024 both use single-gene RT-qPCR workflows for BRSV detection. Similarly, representative laboratory studies such as Zhang et al (Zhang et al., 2022). and Hao et al (Hao et al., 2024). incorporate BRSV as a single-gene target within multiplex RT-qPCR panels, amplifying the N and F genes, respectively. In the present study, we aimed to reduce this theoretical vulnerability by developing a dual-target multiplex RT-qPCR that simultaneously amplifies conserved regions in both the F and N genes. These national standards and published assays collectively indicate that dual-target molecular methods for BRSV detection have been relatively limited in current workflows in China, and their integration into systematic, large-scale epidemiological surveys has not yet been widely implemented. This limited integration in both methodological validation and field application underscores the need for a robust dual-target RT-qPCR assay that can support reliable regional surveillance of BRSV.

Given the pivotal role of BRSV in BRDC and its potential for widespread infection in Inner Mongolia, this study developed and optimized a multiplex RT-qPCR detection method targeting two conserved genes of BRSV. By employing a dual-targeting strategy that simultaneously detects two highly conserved genes, this method provides built-in redundancy and improves analytical robustness of the diagnostic tool. The method was applied to 909 clinical samples collected in Inner Mongolia in 2023, enabling a comprehensive assessment of BRSV prevalence and viral load. This approach provides a methodological foundation and epidemiological data for regional monitoring and targeted control of BRSV.

## 2 Materials and methods

### 2.1 Ethics statement

Sample collection in this study was granted permission by the management of the intensive farms and pastoral households. The experimental protocol was approved by the Experimental Animal Welfare and Ethics Committee of Inner Mongolia Agricultural University. Ethical permission number: NND2022114.

### 2.2 Sample collection

**Figure 1:**
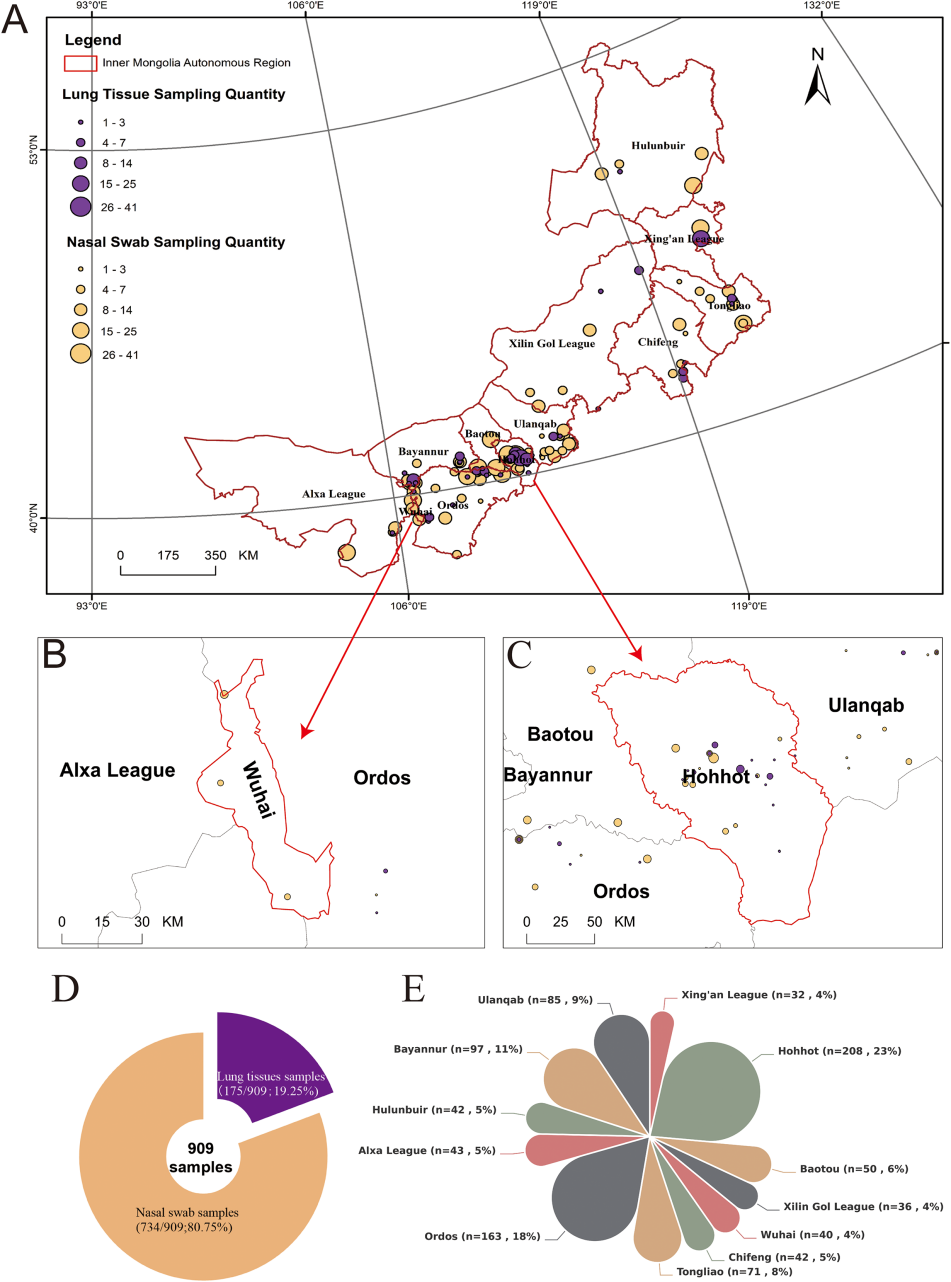
Geographic distribution and sample collection numbers. **(A)** Bubble chart of the geographical distribution of sampling sites. The bubble marks the sampling location, and the size of the bubble reflects the number of samples collected. **(B, C)** provide close-ups of the number of sampling sites in Hohhot and Wuhai, respectively. **(D)** Proportion of nasal swab and lung tissue samples among the 909 samples. **(E)** Proportion of the number of samples collected by each league and city in Inner Mongolia.

From January to December 2023, the research team conducted a structured, clinic-based convenience sampling study on bovine respiratory disease across 12 leagues and cities in the Inner Mongolia Autonomous Region. A total of 909 clinical samples were collected, including 734 nasal swabs and 175 lung tissues. The sample sources covered large-scale cattle farms and free-range farmers, providing broad geographic and management-system coverage (**Figures 1A–E**). All samples were collected by professionally trained local veterinarians using standardized sample-collection procedures, with disposable sterile cotton swabs and tissue sampling cups to ensure consistency of operation and reliability of test results. In order to ensure the quality of samples, they are refrigerated at 4°C immediately after collection, and transferred on ice packs to the laboratory for processing or storage (−80°C) within 72 h prior to testing and analysis. The samples used in this study were all collected from cattle with obvious clinical manifestations at the age of 1 to 6 months. The main clinical symptoms include: fever, serous to purulent nasal secretions, persistent dry and wet coughing, and dyspnea. These manifestations are highly consistent with typical symptoms of BRDC.

This constituted a case-enriched convenience sampling frame representing symptomatic animals presented for veterinary investigation, rather than a random sample of the general cattle population. Therefore, all detection rates reported in this study should be interpreted as case positivity among clinically affected calves. Nasal swabs were collected from live calves at clinical presentation, whereas lung tissues were obtained at necropsy from calves that died or were euthanized due to severe respiratory disease; therefore, specimen types represent different clinical contexts and are not directly comparable as severity-matched samples.

### 2.3 Samples preparation

Nasal swab specimens were centrifuged at 13,000×g for 5 min at 4°C, and 200 μL of the supernatant was transferred to a sterile tube for downstream processing. Nucleic acids were extracted using the Guv Viral DNA/RNA Extraction Kit (GlinX Biotech, China). Multiplex RT-qPCR was performed using Hieff Unicon^®^ Universal Multiplex One-Step Probe qPCR Kit (Yeasen Biological, China). About 20 mg of lung tissue samples were weighed, chopped and homogenized with a tissue grinder. To reduce potential inhibitors from tissue homogenates, nucleic acid extraction was performed using clarified supernatants obtained after centrifugation. The extraction procedure for lung samples was the same as that for nasal swabs. All extracted samples were stored at −80°C for subsequent testing.

### 2.4 Primers and probes

The full-length nucleotide sequences of the F and N genes and bovine proto-oncogene ABL-1 were obtained from the GenBank database. Multiple sequence alignment was conducted using DNASTAR software, and according to the general standards for the design of RT-qPCR primers and probes, highly conserved regions were screened, and specific primers and TaqMan probes were designed based on these regions. To ensure the specificity of the primers, the Primer-BLAST tool from the NCBI database was employed for sequence alignment analysis to avoid nonspecific amplification. All primers and probes were synthesized by Invitrogen (Shanghai) Co., Ltd., and their specific sequences are detailed in **Table 1**.

**Table 1:** Primers and probes designed for multiplex RT-qPCR assay.

Primers/probes	Sequences(5’→3’)	Size(bp)
BRSV-F-F	TATGGGCTAATGGGCAAGAAG	111
BRSV-F-R	TCCCTCCAGGTGTAGTACTTT	
BRSV-F-P	ROX-TGCTACACCACTTGCAATAGCAGATCC-BHQ2	
BRSV-N-F	GGGTGTACTAGCCAAATCAGTC	120
BRSV-N-R	AAACCAGCTTCTCCACCTAAC	
BRSV-N-P	CY5-CAACCTGTTCCATTTCTGCTTGTACGC-BHQ3	
ABL1-F	GGCCTACAACAAGTTCTCCAT	105
ABL1-R	GACAGGTCAATTCCTGGGTAAG	
ABL1-P	HEX-CATCTGGGCATTTGGAGTGTTGCTTTG-BHQ1	
BRSV-F gene -F (Boxus et al., 2005)	AAGGGTCAAACATCTGCTTAACTAG	85
BRSV-F gene -R (Boxus et al., 2005)	TCTGCCTGWGGGAAAAAAG	
BRSV-F gene -P (Boxus et al., 2005)	FAM-AGAGCCTGCATTRTCACAATACCACCCA-BHQ1	
BRSV-N gene -F (Hakhverdyan et al., 2005)	GCAATGCTGCAGGACTAGGTATAAT	124
BRSV-N gene -R (Hakhverdyan et al., 2005)	ACACTGTAATTGATGACCCCATTCT	
BRSV-N gene -P (Hakhverdyan et al., 2005)	FAM-ACCAAGACTTGTATGATGCTGCCAAAGCA-TAMRA-N	

### 2.5 Preparation of positive control plasmid for BRSV

Utilizing the full-length sequences of the F and N genes from the BRSV reference sequence (NC_038272.1) and the partial sequence of ABL-1 (NM_001206860) as templates, plasmids were separately synthesized into the pUC57 vector. The concentrations of the three plasmids were determined, and the copy numbers were calculated. The samples were then diluted to a final concentration of 1×10^9^ copies/μL. Subsequently, an equal volume mixture was prepared with a ratio of F:N:ABL-1:TE=1:1:1:7, followed by a continuous 10-fold serial dilution. The resulting multiplex positive standards were prepared for subsequent experiments.

### 2.6 Primers-probes concentration optimization

The PCR amplification was performed on the CFX96Touch Real-Time Quantitative PCR Detection System (Bio-Rad, USA). The concentrations of primers and probes were optimized by a checkerboard dilution method to 10 μM. The reaction protocol was as follows: reverse transcription at 50°C for 10 min; pre-denaturation at 95°C for 3 min; 95°C for 5 s, 60°C for 30 s for 45 cycles, and fluorescence was collected during the annealing/extension phase.

### 2.7 Sensitivity test and standard curves of the RT-PCR assay

Under the optimized system, the lowest analytical detection limit of the method was validated using triplicate measurements of a multiplex positive standard containing 1×10^6^ to 1×10¹ copies/μL, along with a low-copy sample at 5 copies/μL. A standard curve was constructed using a 10-fold gradient dilution of the multiplex positive standards ranging from 1×10^6^ to 1×10¹ copies/μL. Additionally, RNA extracted from a BRSV-positive clinical specimen was subjected to 10-fold serial dilutions and tested in eight replicates per dilution to evaluate assay performance within a clinical matrix.

### 2.8 Specificity and reproducibility tests

Seven common bovine respiratory pathogens were selected for detection, including: Bovine Viral Diarrhea Virus (BVDV), Infectious Bovine Rhinotracheitis Virus (IBRV), Bovine Parainfluenza Virus Type 3 (BPIV-3), *Mycoplasma bovis*, *Pasteurella multocida* serotype A, *Klebsiella pneumoniae*, and *Mannheimia haemolytica*. Nucleic acids were extracted from all pathogen samples, diluted, and used for detection. A positive control was prepared using a standard sample with 1 × 10^4^ copies/μL, and RNase-free ddH_2_O was used as the negative control. All controls were tested at the same concentration to ensure comparability.

To evaluate the reproducibility of this method, three sets of positive control plasmids with concentrations of 1.0×10^6^, 1.0×10^4^, and 1.0×10¹ copies/μL were selected for analysis. Within each group, each sample was analyzed in triplicate. In addition, the experiment was independently repeated three times at different time points and on different PCR plates across different batches. The average Cq values, standard deviation (SD), and coefficient of variation (CV) were calculated based on the Cq values of the repeated samples, which were used to assess the intra- and inter-group reproducibility of the method. In addition to the plasmid standards, four BRSV-positive clinical samples covering a wide Ct range (Ct 22–37) were also tested in triplicate across three independent runs to further evaluate assay repeatability in real clinical specimens.

### 2.9 Evaluation of ABL1 reference gene stability

A stratified proportional sampling method was employed to extract 40 nasal swabs and 10 lung tissue samples from 909 clinical samples. The RT-qPCR method was applied to detect the housekeeping gene ABL1 in 50 samples to validate its expression stability across different samples.

### 2.10 Comparative evaluation with published singleplex RT-qPCR assay

To further validate the diagnostic performance of the multiplex RT-qPCR assay, a concordance study was conducted using two widely used and peer-reviewed singleplex RT-qPCR assays targeting the F (Boxus et al., 2005) and N genes (Hakhverdyan et al., 2005) of BRSV. From the full dataset of 909 clinical specimens, a total of 200 samples were selected using stratified random sampling to maintain the proportional distribution of specimen types. The dataset consisted of 734 nasal swabs (80.7%) and 175 lung tissues (19.3%); accordingly, 161 nasal swabs and 39 lung tissues were randomly selected using computer-generated random numbers.

**Figure 2:**
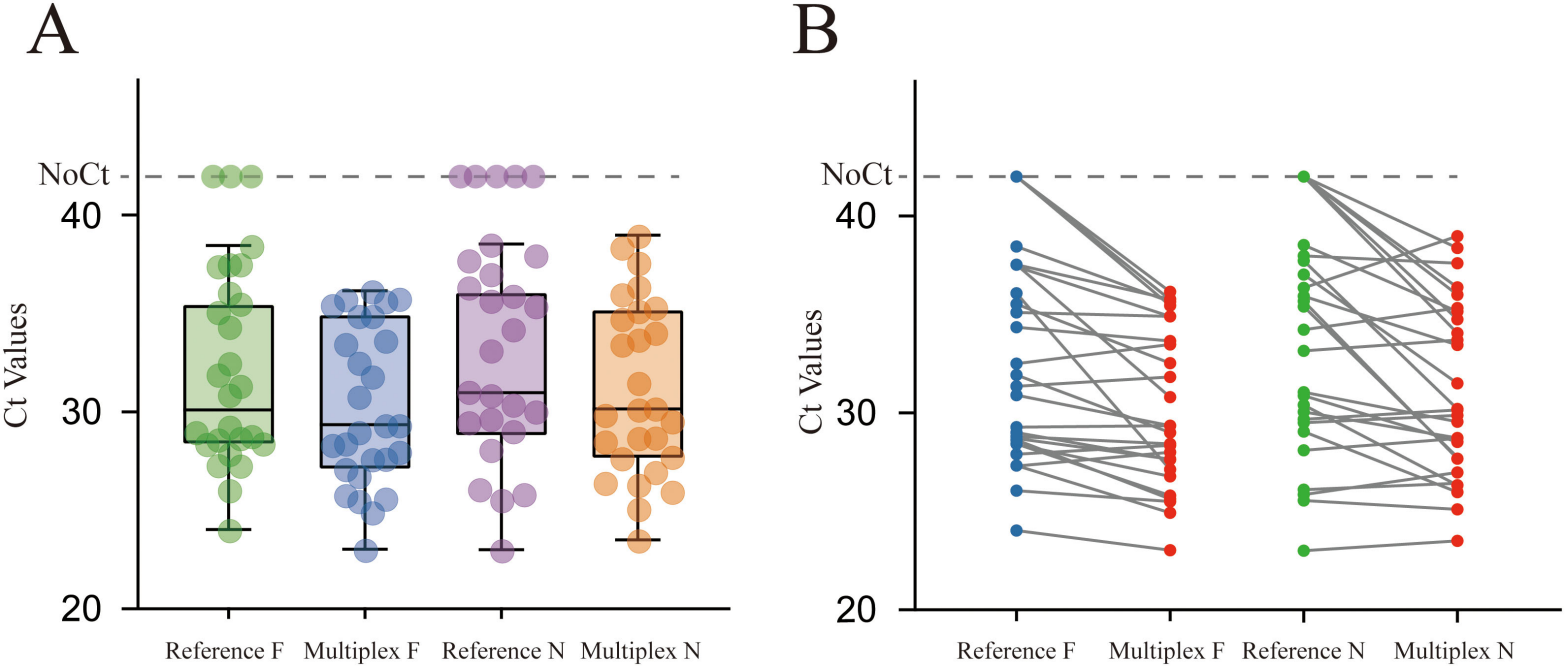
Comparison of the multiplex RT-qPCR assay with published F- and N-gene singleplex assays. **(A)** Ct value distributions for 200 clinical specimens tested in parallel by the multiplex F and N targets and the corresponding reference F and N singleplex RT-qPCR assays. Boxes show the interquartile range with median. **(B)** Paired dumbbell plots comparing Ct values from the multiplex assay with those from the corresponding singleplex assays (left: F gene; right: N gene). For most specimens, Ct differences are within approximately ±2 cycles, indicating high concordance and comparable analytical sensitivity.

All selected samples were tested in parallel using the multiplex RT-qPCR assay and the two published singleplex assays, following the original primer–probe sets and cycling conditions described in the respective publications. For each gene target, positive and negative classifications were recorded, and 2×2 contingency tables were constructed by comparing each target within the multiplex assay with the corresponding published singleplex assay. From these tables, overall, positive and negative agreement were calculated, and Cohen’s kappa (κ) was used to quantify inter-assay agreement beyond chance. These agreement indices are summarized in **Supplementary Table S1**. In addition, internal concordance between the F and N targets within the multiplex assay was assessed by comparing Ct distributions and paired Ct differences, which are illustrated by boxplots and dumbbell plots in **Figure 2**.

### 2.11 Test with clinical samples

All clinical samples were tested using the multiplex RT-qPCR detection method described in this study. A total of 909 samples were processed according to a standardized nucleic acid extraction protocol, and the infection status of BRSV was confirmed based on predefined criteria. The criteria for determination are as follows: samples without fluorescence amplification curves were classified as negative; samples exhibiting amplification curves with Ct values ≤40 were classified as positive; samples with Ct values >40 but still showing amplification curves were categorized as suspicious, necessitating the re-extraction of nucleic acids and repeat testing. If the retest results still showed Ct values >40 with amplification curves, they were ultimately classified as positive. If the internal control does not amplify, re-extraction is required, or the sample is considered invalid. If either the F or N gene was positive, the sample was classified as positive. Samples without Ct values or specific amplification curves were classified as negative.

On this basis, we conducted a systematic epidemiological analysis of the detection results, describing BRSV detection patterns across regions, seasons, production systems and farm-scale categories, and simultaneously assessing viral loads in positive samples. Differences in detection rates between groups were evaluated using Pearson’s chi-square (χ²) tests (or Fisher’s exact test when expected cell counts were < 5), and 95% confidence intervals (95% CI) for proportions were calculated. Viral loads (log^1^^0^-transformed copy numbers) were compared between groups using one-way ANOVA. Together, these analyses provided a comprehensive overview of BRSV epidemiological trends in Inner Mongolia and helped to identify farm- and environment-related risk factors associated with infection.

### 2.12 Statistical analysis

All statistical analyses were performed using SPSS version 27.0 (IBM Corp., Armonk, NY, USA). Graphical visualizations were generated in GraphPad Prism 9.0 and/or R (version 4.4.1). BRSV detection was analyzed as a binary outcome. Detection rates were expressed as percentages with 95% confidence intervals (95% CI), which were calculated using the VassarStats online tool (http://www.vassarstats.net/).

Differences in BRSV detection rates across regions, seasons, specimen types, production systems (intensive farms versus pastoral households) and farm-scale categories were evaluated by Pearson’s chi-square (χ²) tests (Fisher’s exact test when expected cell counts were < 5). The corresponding detection rates, 95% CIs and χ² statistics are reported in **Supplementary Table S2**. To adjust for potential confounding, a multivariable logistic regression model was fitted with BRSV status (positive/negative) as the dependent variable and Region, Season and Farm type as independent variables; adjusted odds ratios (ORs) with 95% CIs are summarized in **Supplementary Table S3**.

For viral-load analyses, F- and N-gene copy numbers were log^1^^0^-transformed before testing. Log^1^^0^ viral loads were compared between groups by one-way analysis of variance (ANOVA) for each combination of gene (F, N) and specimen type (lung tissue, nasal swab), with Region, Season, Farm type or farm-scale category as the factor, as appropriate. When the overall ANOVA was significant, Tukey’s honestly significant difference (HSD) test was used for *post hoc* pairwise comparisons. Unless otherwise stated, all tests were two-sided and P < 0.05 was considered statistically significant.

## 3 Results

### 3.1 Optimization of primers and probes concentrations

After optimizing the primer and probe concentrations using the checkerboard method, the final reaction system composition was as follows: 2×HU^®^ MP Buffer 12.5 μL, HU^®^ Enzyme Mix 1 μL, BRSV-F primers 100 nmol/L, BRSV-F probe 50 nmol/L, BRSV-N primers 100 nmol/L, BRSV-N probe 300 nmol/L, ABL-1 primers and probe 200 nmol/L, Template 5 μL, brought to a final volume of 25 μL with RNase-free ddH_2_O. The reaction protocol was as follows: reverse transcription at 50°C for 10 min; initial denaturation at 95°C for 5 min, 1 cycle; PCR amplification program: 95°C for 15 s, annealing at 60°C for 30 s, for 45 cycles, fluorescence signals were collected during the annealing stage.

### 3.2 Sensitivity and standard curves

To determine the analytical limit of detection, a gradient dilution of multiplex positive standards ranging from 1×10^6^ to 1×10¹ copies/μL, along with a low copy number multiplex positive standard at 5.0 copies/μL, was employed for three repeated measurements to confirm the analytical limit of detection. Based on the limit of detection, criteria for judging the validity of the experimental results were established: the results were considered valid only if both the positive control and the internal reference gene amplification were normal, and the negative control showed no amplification.

**Figure 3:**
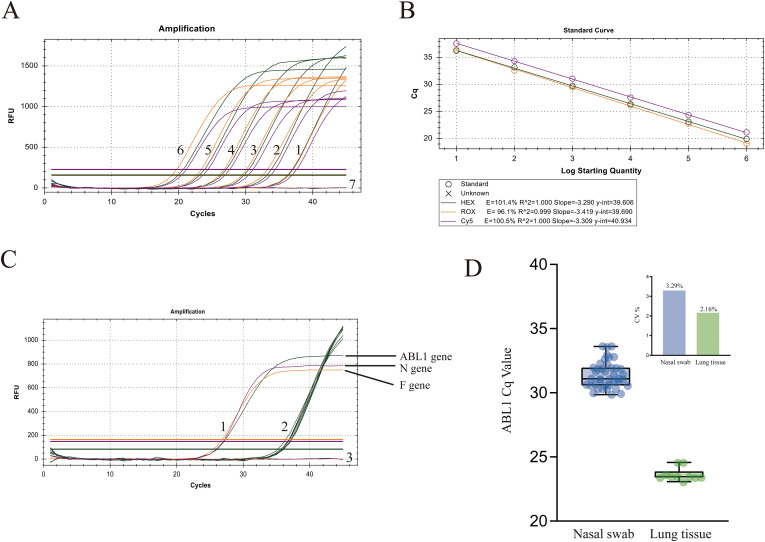
Evaluation of ABL1 reference gene stability and analytical performance of the multi-plex RT-qPCR assay. **(A)** RT-qPCR Amplification Curves 1-6: 1.0×10^1^ to 1.0×10^6^ copies/µL Multi-plex Positive Standard, 7: Negative Control. **(B)** Standard Curve. **(C)** Specificity Detection: 1. 1.0×10^4^ copies/µL Multiplex Positive Standard, 2. Amplification Curve of the Internal Reference Gene for Specificity Detection in the HEX Channel, 3. Negative Controls in the ROX and CY5 Channels and the Nucleic Acids for Specificity Detection. **(D)** Stability evaluation of the ABL1 internal reference gene (Cq values) in nasal swabs and lung tissues; the inset shows the corresponding CV (%).

The results of the standard curve establishment showed (**Figures 3A, B**), for the BRSV F gene, the slope of the standard curve was -3.419, the linear correlation coefficient (R²) was 0.999, the amplification efficiency (E) was 96.1%, and the regression equation was Y=-3.419X+39.690; for the BRSV N gene, the slope was -3.309, R²=1.000, E = 100.5%, and the regression equation was Y=-3.309X+40.934; for the reference gene ABL1, the slope was -3.290, R²=1.000, E = 101.4%, and the regression equation was Y=-3.290X+39.606. The results indicated that the established method had high linearity and reliability, and was suitable for the quantitative detection of BRSV. Based on plasmid standards, the analytical limit of detection for all targets was approximately 5 copies/μL. In this 10-fold serial dilution of clinical RNA, consistent amplification was observed in all replicates up to the 10^-^^4^ dilution, with detectability decreasing at higher dilutions. These results demonstrate the assay’s performance in a clinical matrix (**Table 2**).

**Table 2:** 10-fold serial dilution of BRSV-positive clinical RNA.

Specimen diluted	F gene	N gene	ABL-1 gene
undiluted	24.75 ± 0.10	25.40 ± 0.18	23.08 ± 0.11
1:10	27.42 ± 0.07	28.38 ± 0.13	26.71 ± 0.11
1:100	30.76 ± 0.21	31.63 ± 0.22	29.28 ± 0.28
1:1000	34.16 ± 0.18	35.17 ± 0.42	32.18 ± 0.16
1:10000	36.69 ± 0.54	37.78 ± 0.52	35.45± 0.37

### 3.3 Specificity and reproducibility of the multiplex RT-qPCR assay

To evaluate the specificity of the method, the seven common bovine respiratory pathogens described in Section 2.8 were selected for specific detection. Specific detection was performed using a 1.0×10^4^ copies/μL multiplex positive standard as a positive control and RNase-free ddH_2_O as a negative control. The results revealed (**Figure 3C**) that only the positive control produced a characteristic sigmoidal amplification curve in the ROX and CY5 channels, while no significant amplification was observed for the nucleic acids of the various detected pathogens in both channels, indicating that the method possesses excellent specificity. To further evaluate the repeatability of the method, multiplex positive standards at concentrations of 1.0×10^6^, 1.0×10^4^, and 1.0×10¹ copies/μL were chosen for three repeated tests. The results (**Table 3**) indicate that both intra and inter-batch coefficients of variation were below 2.0%, indicating that the method exhibited good stability and repeatability.

**Table 3:** Repeatability and reproducibility.

Genes of interest	Standard concentrations copies/µL	Intra-group repeatability test		Inter-group repeatability test	
		Mean value(x̄ ± s)	Coefficient of variation(CV/%)	Mean value(x̄ ± s)	Coefficient of variation(CV/%)
BRSV-F	1.0×10^6^	18.81 ± 0.04	0.21	18.98 ± 0.14	0.73
	1.0×10^4^	25.78 ± 0.11	0.44	25.95 ± 0.33	1.28
	1.0×10^1^	36.11 ± 0.51	1.35	36.80 ± 0.68	1.80
BRSV-N	1.0×10^6^	20.12 ± 0.08	0.38	20.08 ± 0.15	0.76
	1.0×10^4^	27.18 ± 0.14	0.52	26.82 ± 0.24	0.90
	1.0×10^1^	36.20 ± 0.41	1.13	36.34 ± 0.43	1.18
ABL1	1.0×10^6^	19.13 ± 0.09	0.46	19.10 ± 0.13	0.66
	1.0×10^4^	25.98 ± 0.13	0.52	25.91 ± 0.18	0.70
	1.0×10^1^	36.32 ± 0.22	0.61	36.50 ± 0.43	1.18

Four BRSV-positive clinical specimens were also tested. As shown in **Table 4**, the intra-assay CV values for BRSV-F and BRSV-N ranged from 0.02% to 1.13%, and the inter-assay CV values ranged from 0.19% to 1.95%, with the internal control ABL1 also exhibiting low variability. Overall, the multiplex RT-qPCR assay showed good repeatability with plasmid controls and maintained relatively stable detection consistency across clinical samples with different viral loads.

**Table 4:** Repeatability of the multiplex RT-qPCR assay in clinical samples.

Genes of interest	Sample name	Intra-group repeatability test		Inter-group repeatability test	
		Mean value(x̄ ± s)	Coefficient of variation(CV/%)	Mean value(x̄ ± s)	Coefficient of variation(CV/%)
BRSV-F	sample-1	27.53 ± 0.01	0.02	27.49 ± 0.15	0.53
	sample-2	31.57 ± 0.08	0.25	31.51 ± 0.21	0.65
	sample-3	37.12 ± 0.25	0.66	37.18 ± 0.38	1.01
	sample-4	24.83 ± 0.10	0.42	24.75 ± 0.17	0.70
BRSV-N	sample-1	28.04 ± 0.04	0.13	28.00 ± 0.13	0.46
	sample-2	31.81 ± 0.27	0.84	31.63 ± 0.19	0.59
	sample-3	36.23 ± 0.41	1.13	35.65 ± 0.70	1.95
	sample-4	25.30 ± 0.06	0.22	25.58 ± 0.05	0.19
ABL1	sample-1	26.57 ± 0.47	1.77	27.29 ± 0.26	0.95
	sample-2	22.95 ± 0.35	1.51	23.28 ± 0.24	1.02
	sample-3	33.10 ± 0.09	0.26	32.75 ± 0.54	1.66
	sample-4	22.21 ± 0.10	0.43	22.25 ± 0.06	0.20

### 3.4 Results of the evaluation of ABL1 reference gene stability

From 909 samples, 40 nasal swabs and 10 lung tissue samples were stratified and extracted for ABL1 expression evaluation of internal control gene stability. The results (**Figure 3D**) showed that 50 samples showed amplification curves, with Cq values concentrated and small fluctuations, and the CV% was below 4%. These findings indicate that ABL1 is stably expressed in nasal swabs and lung tissue samples and can be used as a reliable internal control gene.

### 3.5 Comparative evaluation with published singleplex RT-qPCR assays

Among the 200 stratified randomly selected clinical specimens, the multiplex F/N RT-qPCR assay showed excellent agreement with the two published singleplex assays. For the F gene, the multiplex assay achieved an overall agreement of 98.5% with the reference F singleplex assay, with a positive agreement of 94.1%, a negative agreement of 99.1%, and a Cohen’s kappa (κ) of 0.93. For the N gene, overall agreement with the reference N singleplex assay was 97.5%, with a positive agreement of 89.8%, a negative agreement of 98.6%, and κ = 0.88.

The Ct distributions of the F and N targets in the multiplex assay were highly similar to those obtained with the corresponding singleplex assays (**Figure 2A**), with comparable median values and interquartile ranges and no evident systematic Ct shift. Paired dumbbell plots (**Figure 2B**) further showed that, for most specimens, Ct differences between the multiplex and singleplex assays were within approximately ±2 cycles, with larger discrepancies only in a few high-Ct samples. These findings indicate that the multiplex format does not materially reduce analytical sensitivity and support the robustness and diagnostic consistency of the multiplex F/N RT-qPCR assay for BRSV detection.

### 3.6 Overall of clinical sample testing

This study used an established multiplex RT-qPCR method to detect BRSV and conduct epidemiological analysis of 909 clinical samples collected from Inner Mongolia throughout 2023. The results showed that 193 positive samples were detected, and the overall BRSV detection rate among clinically affected calves was 21.23% (193/909; 95% CI: 18.70% -24.01%). Further analysis of different sample types revealed that, among clinically affected calves, the BRSV detection rate in lung tissue samples was significantly higher than that of nasal swab samples. Among 175 lung tissue samples, 62 were positive, with a positive rate of 35.43% (62/175; 95% CI: 28.73% -42.76%); among 734 nasal swab samples, 131 were positive, with a positive rate of 17.85% (131/734; 95% CI: 15.25% -20.78%).

These values represent case positivity among the sampled cattle and should not be interpreted as population-level prevalence. The internal control gene ABL1 showed stable amplification across all nasal and lung samples, indicating consistent extraction efficiency and negligible inhibition, which supports the reliability of the detection results.

### 3.7 Geographical distribution of BRSV

**Figure 4:**
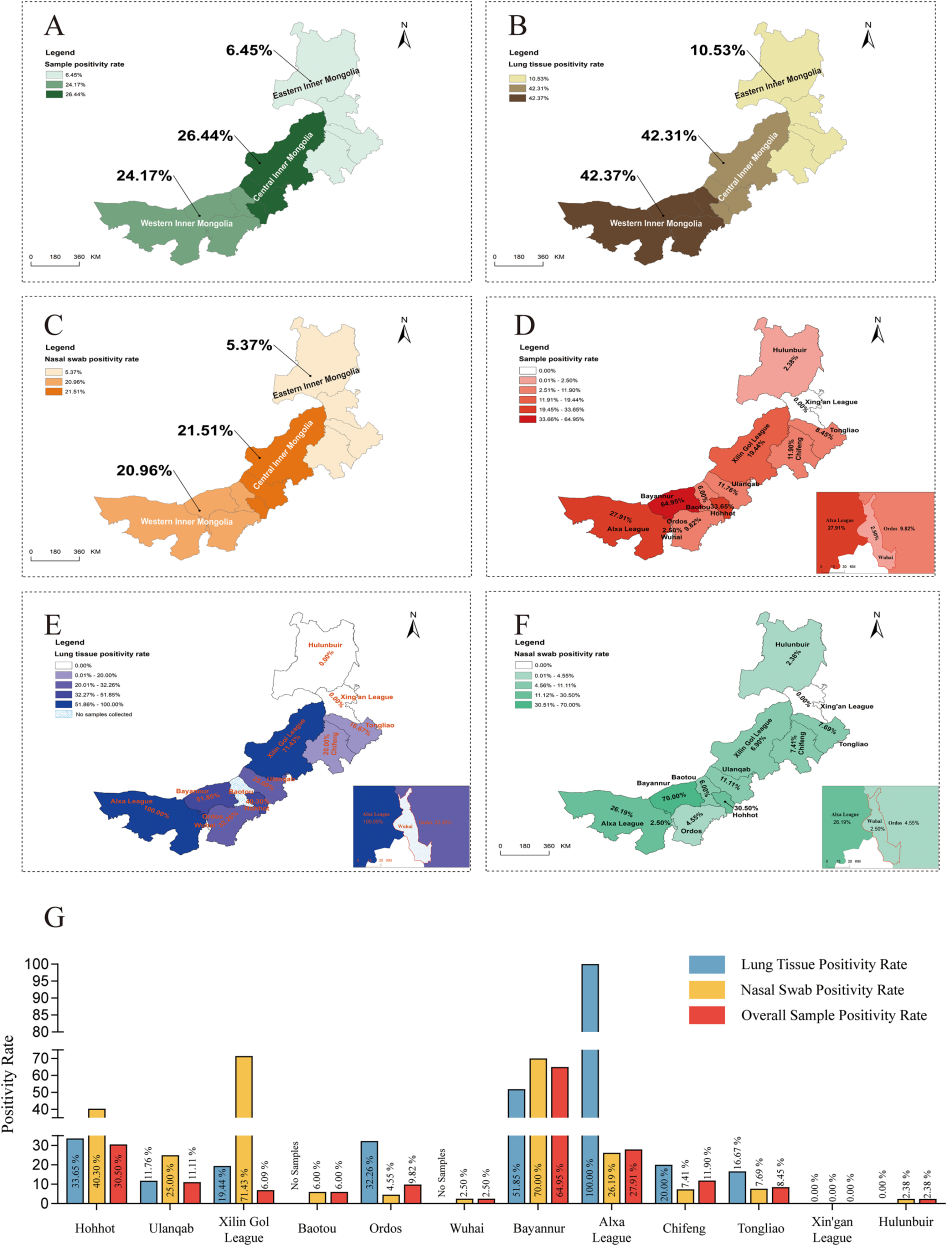
Geographical distribution of BRSV. **(A)** Total positive rate of samples from the eastern, central, and western regions of Inner Mongolia. **(B)** Positive Rate of Lung Tissue Samples from Eastern, Central, and Western Regions of Inner Mongolia. **(C)** Positive Rate of Nasal Swab Sam-ples from Eastern, Central, and Western Regions of Inner Mongolia. **(D)** Total positivity rate of samples from various cities in Inner Mongolia. **(E)** Positive rate of lung tissue samples from vari-ous cities in Inner Mongolia. **(F)** Positive rate of nasal swab samples from various cities in Inner Mongolia. **(G)** Heat map of BRSV-positive rates in various cities in Inner Mongolia.

The regional distribution analysis results (**Figures 4A–C**) showed apparent differences in the detection rate of BRSV among the eastern, central, and western regions of Inner Mongolia. The overall positivity rate in the central region was the highest at 26.44% (87/329), followed by the western region at 24.17% (95/393), and the lowest in the eastern region at only 6.45% (12/186). In lung tissue samples, the positivity rate was highest in the western region at 42.37% (25/59), slightly lower in the central region at 42.13% (33/78), and significantly lower in the eastern region at 10.53% (4/38). In nasal swab samples, the positivity rate was 21.51% (54/251) in the central region, 20.96% (70/334) in the western region, and the lowest in the eastern region at only 5.37% (8/149), based on clinically affected calves with respiratory signs in each region. Because this study used a case-enriched convenience sample, city- and region-level patterns may be influenced by differences in diagnostic submission and specimen composition across locations, and are presented descriptively.

Further analysis of the positivity rates in 12 cities (**Figures 4D–F**) showed that Bayannur had the highest overall positivity rate (64.95%, 63/97), followed by Hohhot (33.65%, 70/208) and Alxa (27.91%, 12/43), while Xing’an (0.00%, 0/32) and Wuhai (2.50%, 1/40) had the lowest, indicating heterogeneity in case positivity among submitted clinical cases across locations among different regions. In lung tissue samples, Alxa (100%, 1/1) and Xilin gol (71.43%, 5/7) had higher rates, while Bayannur (51.85%, 14/27) and Ordos (32.26%, 10/31) were at a medium level; Xing’an (0.00%, 0/17) and Hulunbuir (2.38%, 1/42) had the lowest rates. In nasal swab samples, Bayannur had the highest positive rate among clinically affected calves (70.00%, 49/70), followed by Hohhot (30.50%, 43/141) and Alxa (26.19%, 11/42), Xing’an (0.00%, 0/15), Wuhai (2.50%, 1/40), and Hulunbuir (2.38%, 1/42) had the lowest. These city-level detection rates reflect a case-enriched convenience sample of clinically affected calves submitted for diagnostic investigation in each city, rather than the true population prevalence, and may also be affected by differences in sample submission patterns and the proportion of lung tissue versus nasal swab specimens, particularly where sample sizes were small.

**Figure 4G** summarizes the positivity patterns across the three specimen types for each city, integrates the positivity rates of nasal swabs, lung tissues, and overall samples to visually highlight regional differences. Bayannur showed the highest case positivity across specimen types among submissions, whereas Hohhot and Alxa also exhibited relatively high levels, and Xing’an and Wuhai had almost no detections, consistent with a low-prevalence profile. Statistically, overall BRSV detection differed significantly among the three regions (χ²(2) = 31.19, P = 1.69×10^-7^; **Supplementary Table S2**), and multivariable logistic regression confirmed that, after adjusting for season and farm type, the eastern region remained significantly less affected than the central region (adjusted OR = 0.33, 95% CI: 0.15–0.73; **Supplementary Table S3**).

### 3.8 Temporal distribution of BRSV

**Figure 5:**
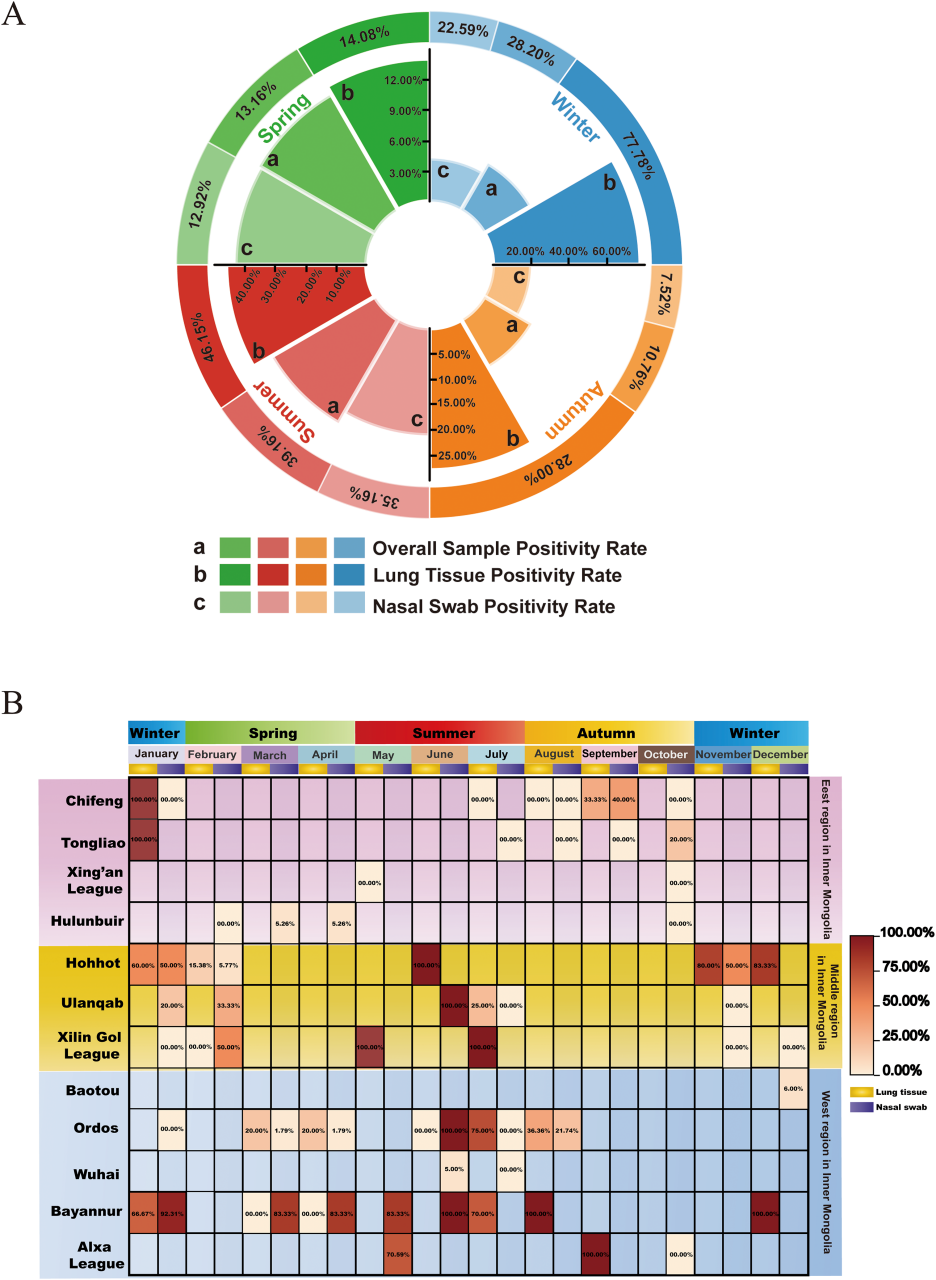
Temporal distribution of BRSV. **(A)** Variation in the overall positive rate of samples, as well as the positive rates of lung tissue and nasal swabs, across four seasons. **(B)** A heatmap illus-trating the overall positivity rates of samples in the eastern, central, and western regions of Inner Mongolia, along with those in various leagues and cities.

This study systematically collected clinical samples from January to December 2023, categorizing the data into four seasons based on the month: spring (February to April), summer (May to July), autumn (August to October), and winter (January, November, and December). The aim of this study was to evaluate the impact of seasonal variations on the detection rate of BRSV. The findings (**Figure 5A**) revealed significant seasonal discrepancies in BRSV detection, with higher positive rates among clinically affected calves observed in summer and winter and lower rates in spring and autumn. The overall positivity rate in May was 50.00% (25/50), the highest for the year. In June, the positivity rate in lung tissue samples reached 80.00% (8/10), higher case positivity among submissions. The positivity rate in July showed a slight decrease. Winter also showed high levels, with lung tissue positivity reaching 90.91% (10/11) in December and 63.64% (7/11) in January. In comparison, the overall detection rates were lower in spring and autumn. For instance, in October, the positivity rate was only 4.00% (3/75), whereas the rates in March and April were 13.49% (17/126) and 14.29% (15/105), respectively. The monthly trends in BRSV transmission showed fluctuations, as illustrated by the difference between 43.14% (44/102) in January and 11.71% (13/75) in February, suggesting that winter viral RNA levels may exhibit periodic variations.

**Figure 5B** further elucidates the seasonal detection patterns of BRSV among clinically affected calves across various regions and months. Overall, significant variations in the BRSV detection rate among clinically affected calves were observed among various prefecture-level cities. The eastern region exhibited generally lower detection rates, with sporadic positive cases reported only in select months. For instance, Chifeng recorded positive detections solely in January and November, whereas Hulunbuir showed a positive rate of 5.26% in March and April, with predominantly negative results in other months, indicating a consistently low prevalence level and sporadic distribution in the eastern region. In contrast, the central region demonstrated markedly higher detection rates with widespread distribution. Hohhot exhibited the highest detection rate levels during summer and winter, with a 100% positive rate in both January and July. Ulanqab also displayed elevated detection rates in summer, reaching 50% in June and 100% in July. Xilin gol presented consistently high levels during both summer and winter, particularly in December, where a 100% positivity rate was observed. These findings suggest the presence of distinct BRSV detection rate peaks in the central region during summer and winter. The western region exhibited higher overall detection rates. Bayannur maintained sustained high levels throughout the year, with peak detection rate observed in January (64.47%), February (52.11%), June (71.43%), July (70.83%), and December (100%), suggesting its potential as a location with persistently high case positivity among diagnostic submissions. Alxa also had elevated detection rates in June (71.43%) and November (100%). Baotou and Ordos exhibited intermediate detection rates, although some months showed positive rates exceeding 30%. In contrast, Wuhai consistently demonstrated the lowest detection rates throughout the year, with positive cases detected in only a few months. Consistent with these patterns, BRSV positivity varied markedly by season (χ²(3) = 58.89, P = 1.01×10^-^¹²; **Supplementary Table S2**), and in the multivariable logistic model, summer had the highest odds of detection compared to autumn (adjusted OR = 3.95, 95% CI: 1.92–8.11), whereas spring showed a significantly lower risk (adjusted OR = 0.45, 95% CI: 0.23–0.90; **Supplementary Table S3**).

In summary, the results indicate that BRSV detection rates among clinically affected calves in Inner Mongolia is characterized by pronounced seasonal fluctuations and significant regional disparities: summer and winter serve as the months/seasons with higher detection among submitted clinical cases, while the central and western regions generally exhibit higher viral RNA levels than the eastern region, with Bayannur displaying the most severe detection rate patterns.

### 3.9 Distribution of BRSV in livestock farms

This study systematically collected clinical samples from different farming models. The samples were categorized based on herd size and farming scale into the following groups: pastoral households (1–100 cattle), small-scale ranches (101–300 cattle), medium-scale ranches (301–1000 cattle), large-scale ranches (1001–3000 cattle), mega-scale ranches (3001–5000 cattle), 10,000-head nucleus farm (5001–10000 cattle), and industrial ranch complexes (over 10,000 cattle).

**Figure 6:**
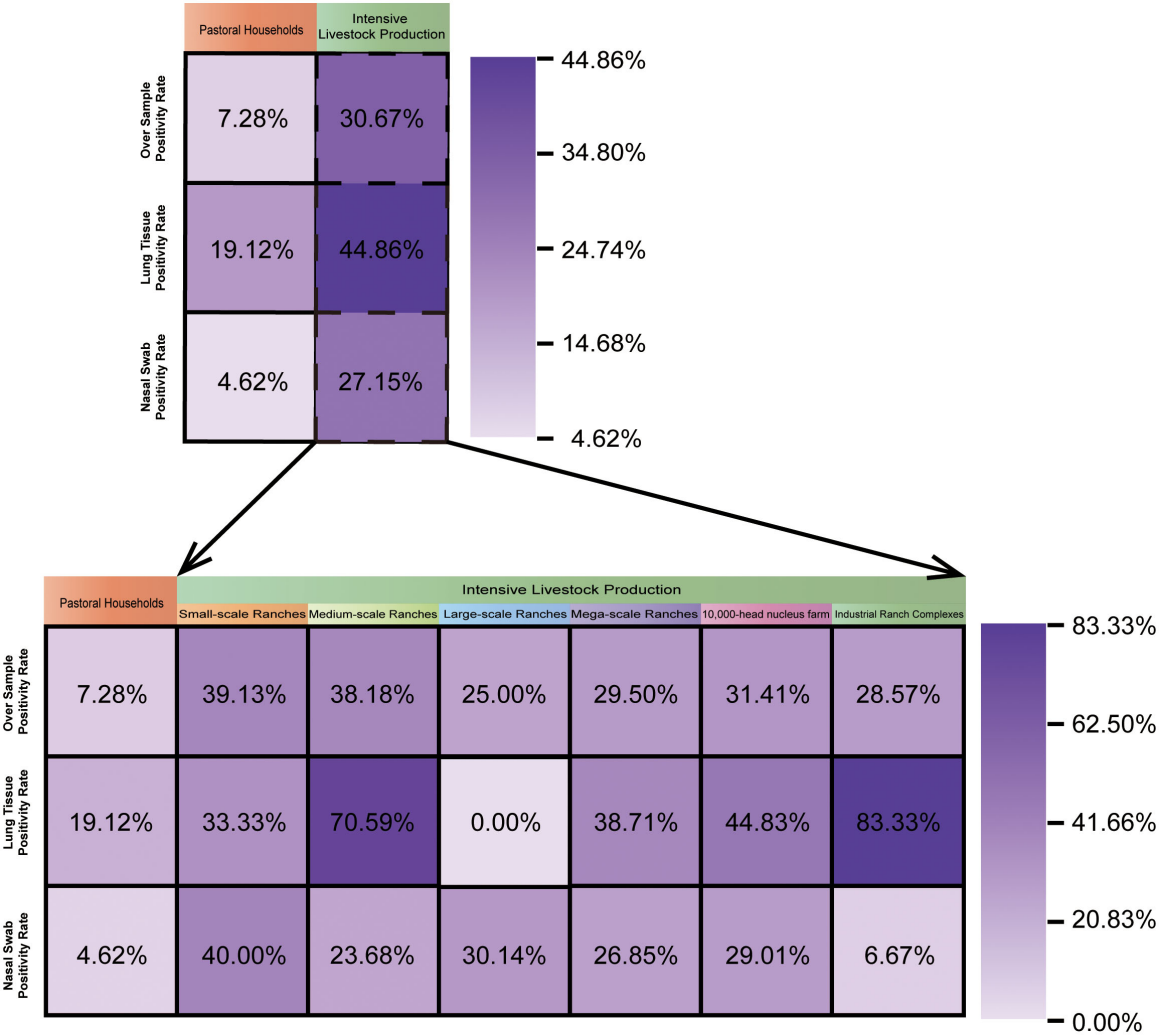
Distribution of BRSV in livestock farms of different scales.

The results (**Figure 6**) showed that among clinically affected calves, the overall BRSV detection rate in pastoral households was only 7.28% (27/371), with a low swab detection rate of 4.62% (14/303), and a lung tissue positive rate of 19.12% (13/68). In contrast, the overall BRSV detection rate among clinically affected calves in intensive farms significantly increased to 30.67% (165/538), with a lung tissue positive rate as high as 44.86% (48/107) and a swab positive rate of 27.15% (117/431), suggesting that the intensive farms may provide more favorable conditions for BRSV transmission in calves with respiratory disease.

In different scales of intensive breeding, among clinically affected calves, the total BRSV detection rate and nasal swab detection rate of the small-scale ranches were the highest, at 39.13% (9/23) and 40.00% (8/20), respectively, and the highest positivity rate of lung tissue in the mega-scale farm cluster was 83.33% (10/12). The overall BRSV detection rates among clinically affected calves in medium-scale ranches and industrial ranch complexes were 38.18% (21/55) and 28.57% (12/42), respectively, but the detection rate of lung tissue was as high as 70.59% (12/17) and 83.33% (10/12), respectively. The nasal swab samples collected from the large-scale ranches were all negative (0.00% (0/15)), and the swab positivity rate of the industrial ranch complexes was only 6.67% (2/30). This distribution suggests that BRSV may be related to differences in breeding density, ventilation conditions, and biosecurity control measures. Statistically, BRSV detection was much more frequent in intensive farms than in pastoral households (30.3% vs 7.7%; χ²(1) = 66.55, P = 3.33×10^-^¹^6^; odds ratio = 5.21, 95% CI: 3.40–7.98; **Supplementary Table S2**). However, among intensive farms alone, detection rates did not differ significantly across the six farm-scale categories (χ²(5) = 3.79, P = 0.58), and the multivariable logistic regression confirmed that production system, rather than herd size, was the main factor associated with detection in this dataset (**Supplementary Table S3**).

### 3.10 Characteristics of viral load

**Figure 7:**
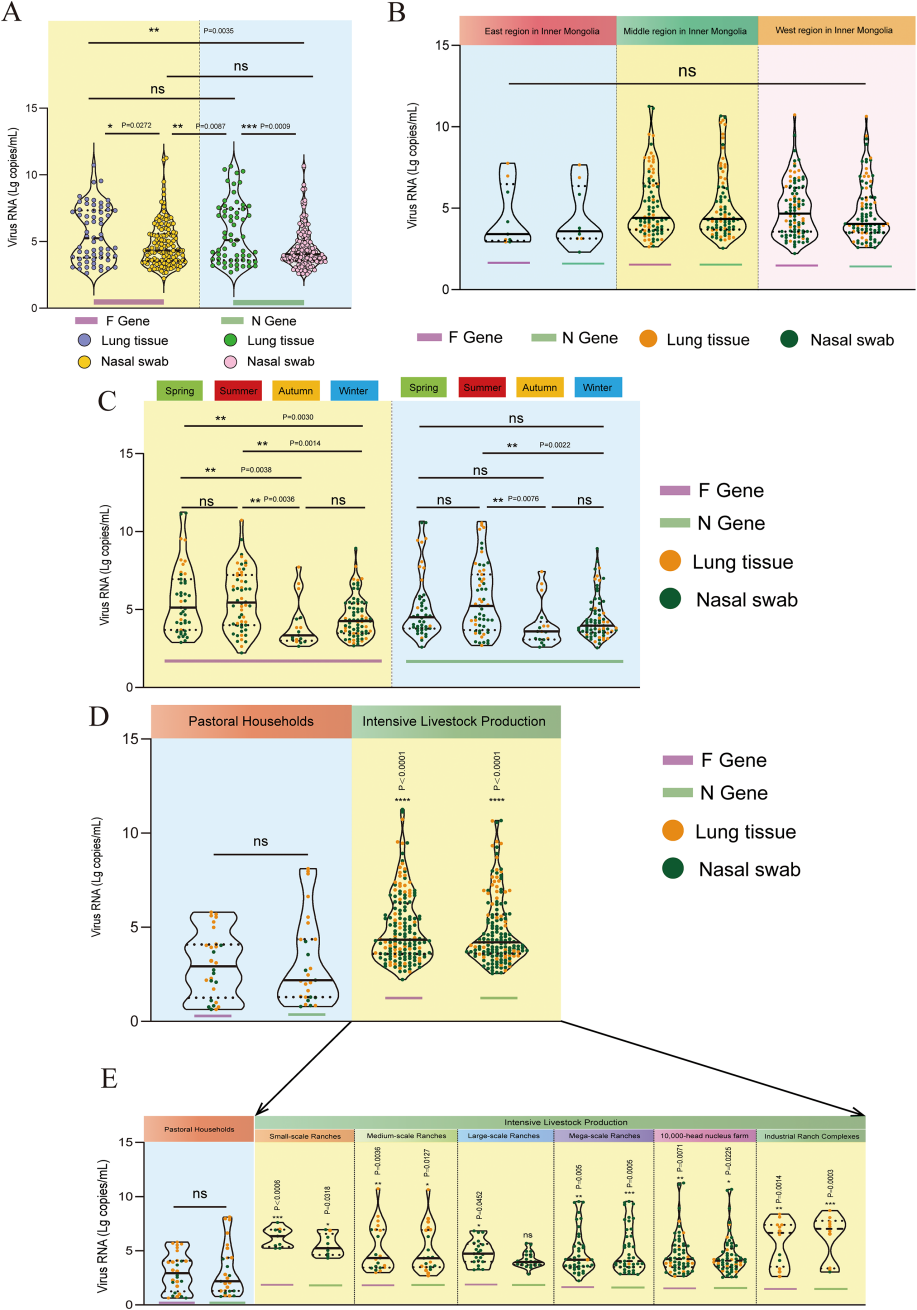
Characteristics of viral load. **(A)** Distribution of viral load of the F and N genes in lung tissues and nasal swab samples. Importantly, lung tissues and nasal swabs were collected from different animals **(B)** Distribution of viral loads in the eastern, central, and western regions of Inner Mongolia. **(C)** Variation trends of viral load across different seasons. **(D)** Distri-bution of viral load between free-range and intensive farming systems. **(E)** Distribution of viral loads across different farm scales. Statistical comparisons were performed using one-way ANOVA followed by Tukey’s HSD test (see **Supplementary Table S4** for full statistics. * indicates P < 0.05, ** indicates P < 0.01, *** indicates P < 0.001, and ns indicates not significant (P ≥ 0.05).

Using the multiplex RT-qPCR methodology developed in this study, viral copy numbers were calculated for 193 samples deemed positive, and the data were subjected to logarithmic transformation for statistical analysis and graphical representation. **Figure 7A** illustrates the distribution of viral loads for the BRSV F and N genes in lung tissue and nasal swab samples. The findings indicate a high degree of similarity in viral load distribution between the F and N genes across both specimen types, suggesting that the multiplex RT-qPCR assay possesses good adaptability and internal consistency. Lung tissue viral loads were higher and more symmetrically distributed than nasal swab loads, with a higher median, indicating more intense viral replication in the lower respiratory tract. In contrast, viral load in nasal swab samples predominantly fell within a lower range, suggesting that the concentration of BRSV RNA in upper respiratory tract samples is typically low. There was a statistically significant difference in viral load between the two sample types, further substantiating the pronounced differences in BRSV viral RNA levels across different sample types. Specifically, for the F gene, the mean log10 viral load was 5.56 in lung tissues versus 4.77 in nasal swabs (one-way ANOVA, F(1,190) = 8.11, P = 0.0049), while for the N gene, mean log10 viral loads were 5.67 and 4.57, respectively (F(1,187) = 14.61, P = 1.8 × 10^-^^4^). Moreover, there were a few individuals in both samples with high viral loads, indicating intense infection in some individuals.

**Figure 7B** illustrates the distribution of viral loads in the eastern, central, and western regions of Inner Mongolia. No significant difference was observed in the viral RNA levels among the three regions. The number of positive samples and viral load in the eastern region were both lower than those in the central and western regions. The viral load in the central region showed a large range of fluctuations, with a bimodal distribution in lung tissues, indicating the existence of high and low load groups. The RNA concentration in nasal swab samples was mainly concentrated in the range of 0 to 5 log copies/mL, which was narrower and lower than in lung tissues. The viral load in the western region was slightly higher than that in the eastern region but lower than that in the central region. The viral concentration in most lung tissue samples in the western region was concentrated in the range of 5–10 log copies/mL, suggesting moderate and concentrated infection intensity in this region. Consistent with these visual patterns, one-way ANOVA showed no significant regional differences in viral load for either gene or specimen type (all P ≥ 0.33; **Supplementary Table S4**).

**Figure 7C** illustrates seasonal fluctuations in viral load, with higher RNA concentrations in spring and summer and a peak in summer. The RNA concentrations of the F and N genes were significantly higher in spring and summer than in autumn and winter. Lung tissue samples showed markedly higher viral loads in spring and summer than in autumn and winter, and nasal swab samples exhibited a similar pattern, with the highest concentrations in summer. In autumn and winter, viral loads were lower and less variable. Although the overall viral concentration in nasal swabs remained lower than that in lung tissues, seasonal fluctuations indicated active upper respiratory infections in spring and summer. In contrast, viral loads were relatively lower and less variable during autumn and winter, particularly in winter, when overall levels were stable and relatively subdued. In contrast, seasonal effects on viral load were significant (e.g., F gene lung: F(3,58) = 6.24, P = 0.0010; F gene nasal swabs: F(3,126) = 4.83, P = 0.0032), with Tukey’s HSD indicating higher loads in spring and summer than in winter and the lowest loads in autumn for nasal swabs (**Supplementary Table S4**).

**Figure 7D** contrasts the viral load across different farming paradigms, while **Figure 7E** further depicts viral RNA levels for F and N genes across herd-size categories within the intensive production system. Viral loads were significantly lower in pastoral households than in intensive farms (Tukey’s HSD, adjusted P < 0.0001). Within the six herd-size categories of intensive livestock production, viral loads in all intensive farm categories were higher than in pastoral households (adjusted P range: 0.0452 to <0.0001). Several significant contrasts were also observed among intensive categories themselves (for example, some comparisons between small-scale and medium-scale or between medium-scale and mega-scale ranches; **Supplementary Table S4**), suggesting a positive association between production intensity and viral replication. Collectively, these data indicate that both farming system and herd size significantly affect BRSV viral load, and that sampling site (lung tissue vs nasal swab) is an important determinant for accurate quantification though these were from different animals.

## 4 Discussion

Bovine Respiratory Disease (BRD) is a significant disease that poses a severe threat to the cattle industry, resulting in substantial economic losses (Bell et al., 2021). BRD typically results from synergism, including BVDV, BoHV-1, BPIV-3 and BRSV, together with bacteria such as *M. haemolytica*, *P. multocida* and *M. bovis* (McGill et al., 2016; Oguejiofor et al, 2019; Gaudino et al., 2022). Among them, BRSV is one of the key pathogens of BRD and is frequently co-infected with other pathogens, thereby elevating morbidity and mortality rates and exacerbating control and treatment costs. Therefore, early diagnosis and detection of BRSV hold significant economic and clinical importance (Zhang et al., 2022). We developed a dual-target RT-qPCR method for BRSV F and N genes, incorporating the Bos taurus ABL proto-oncogene 1 as an internal control to monitor sample extraction and amplification quality. After optimization, the method achieved specific amplification of the F, N, and ABL1 genes, with CVs below 2.0%, demonstrating excellent stability and reproducibility. Although RNA purity and integrity were not directly assessed, the stable amplification of the internal control gene ABL1 across nasal and lung samples indicates that potential inhibitors from lung tissue had minimal impact on RT-qPCR performance. We compared the new assay with two widely applied published singleplex RT-qPCR assays targeting the BRSV F gene (Boxus et al., 2005) and the N gene (Hakhverdyan et al., 2005). A stratified random sample of 200 representative clinical specimens was selected from the full dataset of 909 samples. The new assay performed comparably to established singleplex methods. Internal concordance analysis of the multiplex assay showed that the F and N targets were highly consistent (99.5%), with only one discordant sample (F^+^/N^-^) identified among the 200 tested specimens. Insufficient RNA remained for sequencing to resolve this discrepancy; it remains unclear whether mutations in the F- and N-gene target regions or an extremely low template concentration contributed to the result.

The limit of detection (≈5 copies/μL) was determined using plasmid standards and reflecting performance under ideal conditions. It is important to emphasize that plasmid DNA does not fully replicate the properties of viral RNA extracted from clinical specimens, which can differ in RNA integrity, extraction efficiency, and potential PCR inhibition. Consequently, the analytical LOD may not reflect clinical sensitivity. The results of a 10-fold serial dilution experiment using a BRSV-positive clinical specimen, with eight replicates per dilution, showed consistent detection across dilutions, though Ct variability increased at higher dilutions. These findings indicate that the assay can detect low-abundance BRSV RNA in naturally infected material and provide complementary evidence to the plasmid-based analytical sensitivity assessment.

In this study, all sampled animals were clinically affected calves. The survey showed that the BRSV detection rate among clinically ill calves in Inner Mongolia was 21.23% (193/909) (95% CI: 18.70%–24.01%), indicating that BRSV was frequently detected in this case-enriched BRDC cohort. Geographically, the detection rate of BRSV was notably higher in the central and western regions of Inner Mongolia than in the eastern region, with Bayannur ranking among the top positive rates. These extreme values (e.g., 0% in Xing’an, 64.95% in Bayannur) must be interpreted cautiously, as they may reflect differences in sample submission patterns and farm types, not just true prevalence. This discrepancy may be attributed to the higher livestock density, frequent cattle movement, and potentially inadequate implementation of biosecurity measures in the central and western regions. In contrast, the eastern region, with a smaller scale of farming and more dispersed cattle populations, exhibits a lower detection rate, characterized by sporadic outbreaks. Seasonally, BRSV detection rates among clinically affected calves were higher in summer and winter, suggesting a bimodal distribution that may be influenced by regional climatic and management conditions. In 2023, the number of high-temperature days at or above 35°C increased by 1.9 days compared with the long-term average, and total summer rainfall reached 199.9 mm, leading to higher ambient humidity. Under semi-enclosed housing with limited ventilation, such conditions may intermittently expose cattle to combined heat and humidity stress, which could reduce feed intake, weaken mucosal immunity, and may increase the risk of respiratory infection (Louie et al., 2018; Cartwright et al., 2023). During the extended cold season, when the mean temperature was −11.7°C, cattle are generally confined indoors to maintain warmth, often under conditions of poor air circulation. Cold stress together with ammonia accumulation may facilitate viral transmission (Gorden and Plummer, 2010; Stokstad et al., 2020). These climatic and management factors coincided with increased BRSV detection, though causality cannot be established.

It is therefore plausible that seasonal environmental stressors contribute to the observed bimodal trend, a hypothesis that warrants confirmation through long-term monitoring and controlled experimental studies. The mode and scale of livestock farming also significantly influenced the BRSV detection rate among clinically affected calves. Intensive farming systems showed a significantly higher detection rate of BRSV than free-range systems, with large and medium-sized ranches having high positive rates in lung tissue samples. The high density of cattle and poor air circulation facilitate virus transmission, suggesting that large ranches should be prioritized in prevention efforts. Improving ventilation conditions and optimizing population density management can help reduce the risk of infection. The chi-square and logistic regression analyses consistently showed that regional and seasonal variations in BRSV detection were statistically significant and remained stable after controlling for farm scale. Viral load was influenced by sample type, season, and farming practice. In terms of sample types, the viral RNA levels in lung tissue samples were significantly higher than those in nasal swabs, suggesting that the lungs are the consistent with lower-respiratory-tract tropism of BRSV. Conversely, the viral loads in nasal swabs were generally lower, in alignment with the biological characteristics of BRSV, which predominantly replicates in the lower respiratory tract Nasal swab viral loads were generally lower, consistent with BRSV’s tropism for the lower respiratory tract (Sudaryatma et al., 2019). however, nasal swabs and lung tissues were collected from different animals in this study, so direct comparisons should be interpreted cautiously. In terms of regional distribution, no statistically significant differences were observed in viral loads among the eastern, central, and western regions of Inner Mongolia. By contrast, viral load was significantly lower under extensive farming conditions, whereas RNA levels in samples from intensive farming practices were markedly elevated, showing a highly significant difference (*P < 0.0001*). Further stratification showed that large and mega scale farms had significantly higher viral loads than small and medium-sized ranches. However, an unusual pattern was observed. Specifically, in several of these herds, the average viral RNA levels detected in nasal swabs were higher than those in lung tissues. It should be clarified that the nasal swab and lung tissue samples analyzed in this study were obtained from different animals, rather than paired samples from the same individuals. In addition, lung tissues were more likely to originate from the most severe cases undergoing necropsy, whereas nasal swabs were collected from live clinical presentations; therefore, between-specimen viral-load differences may be partly confounded by disease severity or stage and should be interpreted cautiously.

The seasonal pattern of viral RNA levels among BRSV-positive samples is not identical to the seasonal pattern of BRSV detection rates among clinically affected calves. Detection rates, which peaked in summer and winter, describe the proportion of clinically affected calves in this case-enriched sample that tested positive for BRSV in each season. By contrast, the viral load analysis is restricted to BRSV-positive samples and reflects the distribution of viral RNA levels within infected animals, in which median viral loads tended to be higher in spring and summer than in autumn and winter. Because we did not collect standardized clinical severity scores, time-since-onset information or detailed herd-level outbreak data, we cannot determine whether these patterns reflect differences in infection stage or disease severity across seasons. Therefore, seasonal viral load differences are treated as descriptive and hypothesis-generating. In light of this case-enriched convenience sampling of clinically affected calves, the viral load values reported in this study should therefore be interpreted primarily as relative indicators of BRSV viral RNA abundance across sample types, seasons and farming systems, rather than as validated prognostic thresholds or direct measures of infectiousness.

In summary, we developed a sensitive and specific dual-gene multiplex RT-qPCR assay for BRSV and enhanced its reliability and applicability across different sample types by incorporating the reference gene ABL1. In conjunction with epidemiological data analysis, this study described epidemiological characteristics and viral load differences of BRSV across different regions of Inner Mongolia, seasons, and farming systems. BRSV circulates widely among clinically ill calves in Inner Mongolia and is influenced by multiple factors. It should also be acknowledged that this investigation was based on a convenience sample of clinically affected calves rather than a probability-based survey of the general cattle population. This convenience sampling framework may over-represent herds with more severe clinical signs or better access to veterinary diagnostic services and under-represent subclinical or mildly affected animals, introducing selection and spectrum bias. A further limitation is that concurrent, panel-based testing for other major BRD pathogens was not performed, preventing assessment of BRSV’s specific role versus co-infections. This study was intentionally limited to BRSV; therefore, concurrent panel-based testing for other BRDC pathogens was not performed, and our findings should be interpreted as BRSV detection and viral-load patterns within a clinically affected cohort. Future studies incorporating standardized multi-pathogen diagnostic panels will be required to quantify the joint and relative effects of BRSV and co-pathogens on clinical outcomes and viral load dynamics.

## 5 Conclusions

This study developed and validated a multiplex RT-qPCR detection method targeting two genes of BRSV. This method was applied to test 909 clinical samples from calves with respiratory signs in Inner Mongolia, and the results demonstrated that the assay showed excellent sensitivity, specificity, and stability, providing indicative viral load data. The assay is promising for surveillance, revealing spatial, temporal, and management-related infection patterns. The assay shows potential for case-based detection, though further validation is needed for broader use. In particular, longitudinal validation in sentinel herds including both clinically healthy and symptomatic animals will be important to establish baseline positivity and incidence.

## Data Availability

The raw data supporting the conclusions of this article will be made available by the authors, without undue reservation.
