# Supplementary material for: Molecular investigation and viral load analysis of bovine respiratory syncytial virus in cattle with bovine respiratory disease complex in Inner Mongolia, China
Source: Front Cell Infect Microbiol. 2026 Feb 13;16:1720214. doi: 10.3389/fcimb.2026.1720214 (PMC12946127; doi:10.3389/fcimb.2026.1720214)
Supplement: Supplementary file 1 [file Table1.docx]

**Supplementary Table S1.**

**(A) 2×2 contingency tables**

| **Target** | **Reference assay result** | **Multiplex positive** | **Multiplex negative** | **Total** |
| --- | --- | --- | --- | --- |
| **F gene** | Positive | 24 | 0 | 24 |
|  | Negative | 3 | 173 | 176 |
|  | Total | 27 | 173 | 200 |
| **N gene** | Positive | 22 | 0 | 22 |
|  | Negative | 5 | 173 | 178 |
|  | Total | 27 | 173 | 200 |

**(B) Agreement indices**

| **Target** | **Overall**  **agreement (%)** | **Positive**  **agreement (%)** | **Negative**  **agreement (%)** | **Cohen’s κ** |
| --- | --- | --- | --- | --- |
| **F gene**  **(multiplex F vs reference F singleplex)** | 98.5 | 94.1 | 99.1 | 0.93 |
| **N gene**  **(multiplex N vs reference N singleplex)** | 97.5 | 89.8 | 98.6 | 0.88 |

Agreement between the multiplex F/N RT-qPCR assay and published singleplex RT-qPCR assays targeting the F and N genes of BRSV (n = 200). Overall agreement = (true positives + true negatives) / total; Positive agreement and Negative agreement are calculated from the 2×2 tables for each gene; Cohen’s κ quantifies agreement beyond chance.

**Supplementary Table S2.**

| **Grouping variable** | **Category** | **Total samples (n)** | **BRSV-positive (n)** | **Positivity (%)** | **95% CI for proportion (%)** |
| --- | --- | --- | --- | --- | --- |
| **Sample type** | Lung tissue | 175 | 62 | 35.4 | 28.7 – 42.8 |
|  | Nasal swab | 734 | 131 | 17.9 | 15.3 – 20.8 |
| **Region** | Central | 330 | 87 | 26.4 | 21.9 – 31.4 |
|  | Western | 393 | 94 | 23.9 | 20.0 – 28.4 |
|  | Eastern | 186 | 12 | 6.5 | 3.7 – 10.9 |
| **Season** | Spring | 342 | 45 | 13.2 | 10.0 – 17.2 |
|  | Summer | 143 | 56 | 39.2 | 31.5 – 47.3 |
|  | Autumn | 158 | 17 | 10.8 | 6.8 – 16.6 |
|  | Winter | 266 | 75 | 28.2 | 23.1 – 33.9 |
| **Production system** | Intensive livestock production | 545 | 165 | 30.3 | 26.6 – 34.3 |
|  | Pastoral households | 364 | 28 | 7.7 | 5.4 – 10.9 |
| **Farm scale (intensive farms only)** | Small-scale ranches | 23 | 9 | 39.1 | 22.2 – 59.2 |
|  | Medium-scale ranches | 55 | 21 | 38.2 | 26.5 – 51.4 |
|  | Large-scale ranches | 88 | 22 | 25 | 17.1 – 35.0 |
|  | Mega-scale ranches | 139 | 41 | 29.5 | 22.5 – 37.6 |
|  | 10,000-head nucleus farms | 191 | 60 | 31.4 | 25.3 – 38.3 |
|  | Industrial ranch complexes | 42 | 12 | 28.6 | 17.2 – 43.6 |

Chi-square tests (Pearson’s χ², two-sided):

Sample type (lung vs nasal): χ²(1) = 26.12, P = 3.2 × 10⁻⁷

Region (central vs western vs eastern): χ²(2) = 31.19, P = 1.7 × 10⁻⁷

Season (spring vs summer vs autumn vs winter): χ²(3) = 58.89, P = 1.0 × 10⁻¹²

Production system (intensive vs pastoral): χ²(1) = 66.55, P = 3.3 × 10⁻¹⁶; odds ratio (intensive vs pastoral) = 5.21 (95% CI: 3.40 – 7.98)

Farm scale (within intensive farms): χ²(5) = 3.79, P = 0.58

Note:

All 95% confidence intervals (95% CI) for proportions were calculated using the Wilson method (as implemented in the VassarStats online calculator).

**Supplementary Table S3**

Multivariable logistic regression analysis of factors associated with BRSV detection (n = 909).

| **Predictor** | **Category** | **Reference category** | **Adjusted OR** | **95% CI for OR** | **P-value** |
| --- | --- | --- | --- | --- | --- |
| **Region** | Eastern | Central | 0.33 | 0.15 – 0.73 | 0.006 |
|  | Western | Central | 0.98 | 0.65 – 1.50 | 0.939 |
| **Season** | Spring | Autumn | 0.45 | 0.23 – 0.90 | 0.024 |
|  | Summer | Autumn | 3.95 | 1.92 – 8.11 | 0.00018 |
|  | Winter | Autumn | 1.9 | 0.92 – 3.95 | 0.085 |
| **Farm type** | Pastoral households | Intensive livestock production | 0.12 | 0.08 – 0.19 | <0.0001 |

Outcome variable: BRSV status (1 = positive, 0 = negative) as determined by the multiplex F/N RT-qPCR assay.

Adjusted odds ratios (OR) and 95% confidence intervals are derived from a logistic regression model including Region, Season, and Farm type as predictors.

An OR < 1 indicates lower odds of BRSV detection relative to the reference category; an OR > 1 indicates higher odds.

Model diagnostics (goodness-of-fit and multicollinearity checks) did not indicate major violations of model assumptions.
